# Pathophysiology of right ventricular failure in acute pulmonary embolism and chronic thromboembolic pulmonary hypertension: a pictorial essay for the interventional radiologist

**DOI:** 10.1186/s13244-019-0695-9

**Published:** 2019-02-13

**Authors:** Yolanda C. Bryce, Rocio Perez-Johnston, Errol B. Bryce, Behrang Homayoon, Ernesto G. Santos-Martin

**Affiliations:** 1 Radiology Department, Memorial Sloan Kettering Cancer Center, 1275 York Ave, New York, NY 10065 USA; 2 Internal Medicine, Health Science Center, University of North Texas, 1622 8th Ave, Suite 110, Fort Worth, TX 76104 USA; 3 Radiology Department, University of British Columbia, 13750 96th Ave, Surrey, BC V3V 1Z2 Canada

**Keywords:** Right ventricular failure, Submassive pulmonary embolism, Massive pulmonary embolism, Chronic thromboembolic pulmonary hypertension

## Abstract

Pulmonary embolus (PE) is the third most common cause of cardiovascular death with more than 600,000 cases occurring in the USA per year. About 45% of patients with acute PE will have acute right ventricular failure, and up to 3.8% of patients will develop chronic thromboembolic pulmonary hypertension (CTEPH) with progressive, severe, chronic heart failure. The right ventricle (RV) is constructed to accommodate a low-resistance afterload. Increases in afterload from acute massive and submassive PE and CTEPH may markedly compromise the RV function leading to hemodynamic collapse and death. The purpose of this educational manuscript is to instruct on the pathophysiology of RV failure in massive and submassive PE and CTEPH. It is important to understand the pathophysiology of these diseases as it provides the rationale for therapeutic intervention by the Interventional Radiologist. We review here the pathophysiology of right ventricular (RV) failure in acute massive and submassive PE and CTEPH.

## Teaching points

The anatomic construct of the right heart is suitable for its task. The right ventricle (RV) as part of a low-pressure system with a low-resistance afterload (the pulmonary artery) is thin-walled, compliant, and crescent-shaped.

With increases in afterload, the right ventricle cannot unload sufficiently resulting in dilatation of the compliant right ventricle, impinging on the left ventricle resulting in decreased left ventricle output and supply to the coronary arteries.

Massive pulmonary embolus (PE) is defined as PE with sustained hypotension (systolic BP < 90 for at least 15 min), need for inotropic support, or persistent bradycardia (HR < 40 bpm with signs or symptoms of shock).

Patients with submassive PE are systemically normotensive with evidence of myocardial dysfunction and ischemia.

In chronic thromboembolic pulmonary hypertension (CTEPH), RV dilatation and wall hypertrophy increase oxygen demand to which the coronary artery blood flow cannot meet, resulting in ischemia, necrosis, and fibrosis of the RV wall.

## Introduction

PE is the third most common cause of cardiovascular death (after myocardial infarction and stroke), and more than 600,000 cases are believed to occur in the USA per year [1]. About 45% of patients with PE will have right ventricular compromise, carrying a mortality of up to 25% when the patient is normotensive and up to 65% in the setting of hypotension [2]. Moreover, up to 3.8% of patients with PE develop chronic thromboembolic pulmonary hypertension (CTEPH), a long-term progressive complication of acute PE leading to severe heart failure and death [3, 4]. Understanding the pathophysiology of RV heart failure in these diseases formulates the rationale for therapeutic intervention by the interventional radiologist.

## Differences in the right and left heart

**Fig. 1:**
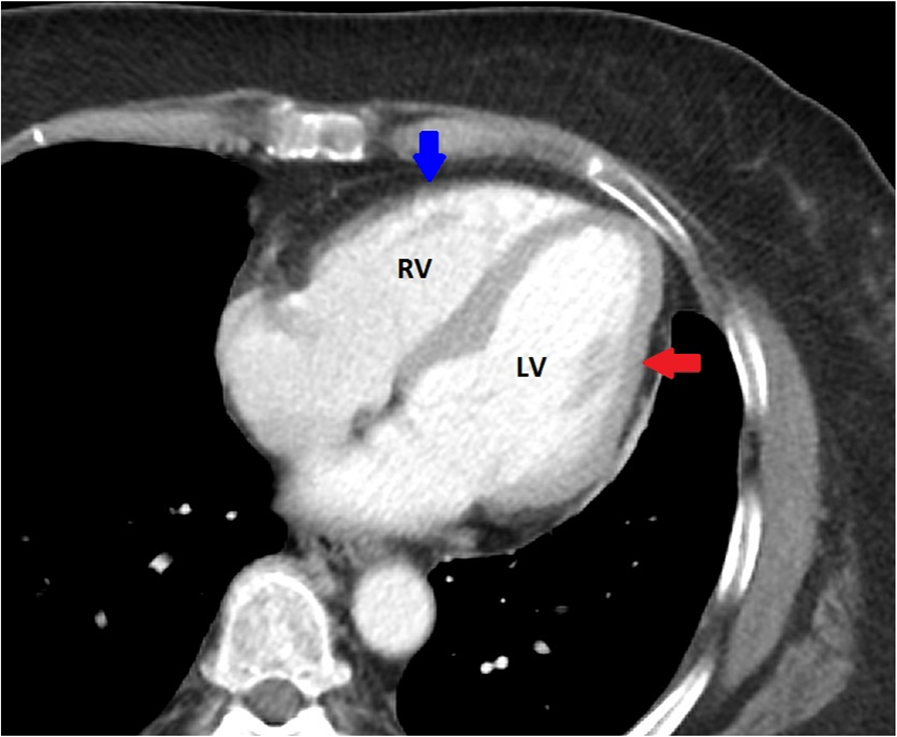
Right and left heart. Blue arrow demonstrates the right ventricle with a thin wall and crescent shape. Red arrow demonstrates the thicker wall of the left ventricle

**Fig. 2:**
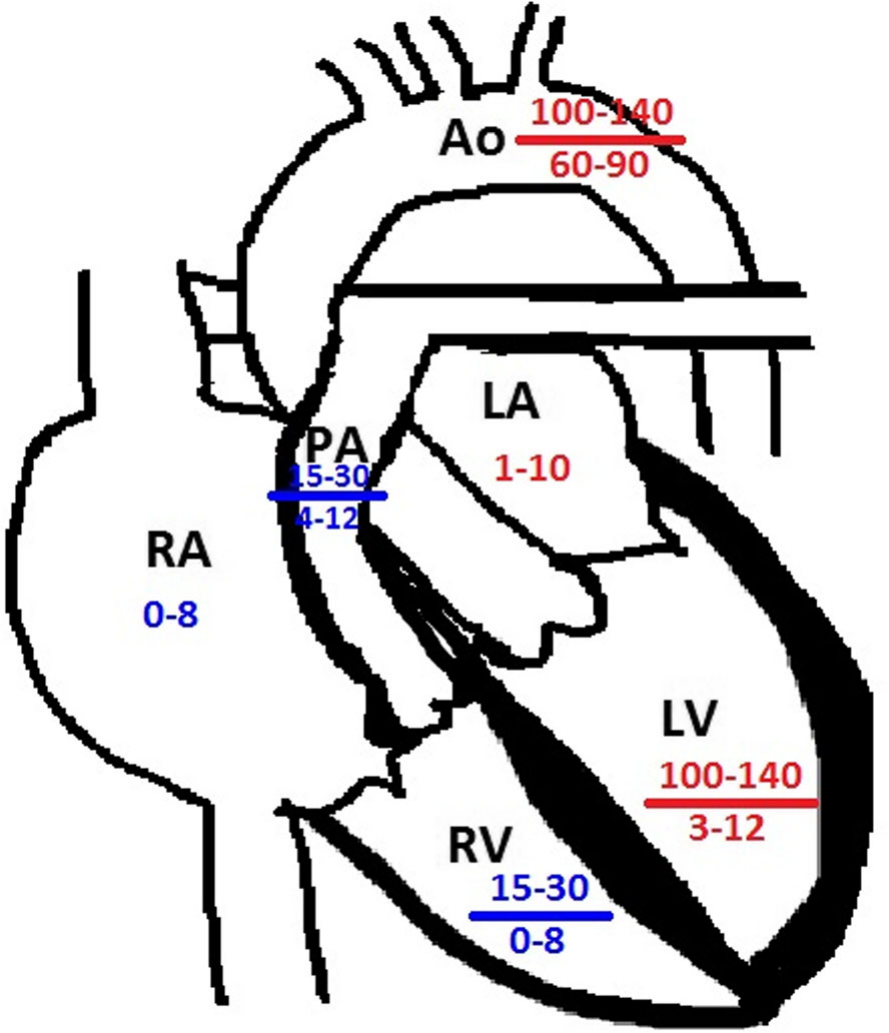
Right and left heart pressures. Diagram demonstrates the right (blue) and left (red) heart pressures

The anatomic construct of the right heart and the left heart are suitable for their task. The RV is part of a low-pressure system with a low-resistance afterload (the pulmonary artery), and is thin-walled, compliant, and crescent-shaped (Fig. 1), able to accommodate a large amount of blood, but without a lot of pressure generated (see Fig. 2) [5]. The left ventricle (LV), on the other hand, is thick-walled (Fig. 1) and non-compliant generating a large amount of pressure against a high-resistance afterload (the aorta) (Fig. 2) [5].

**Fig. 3:**
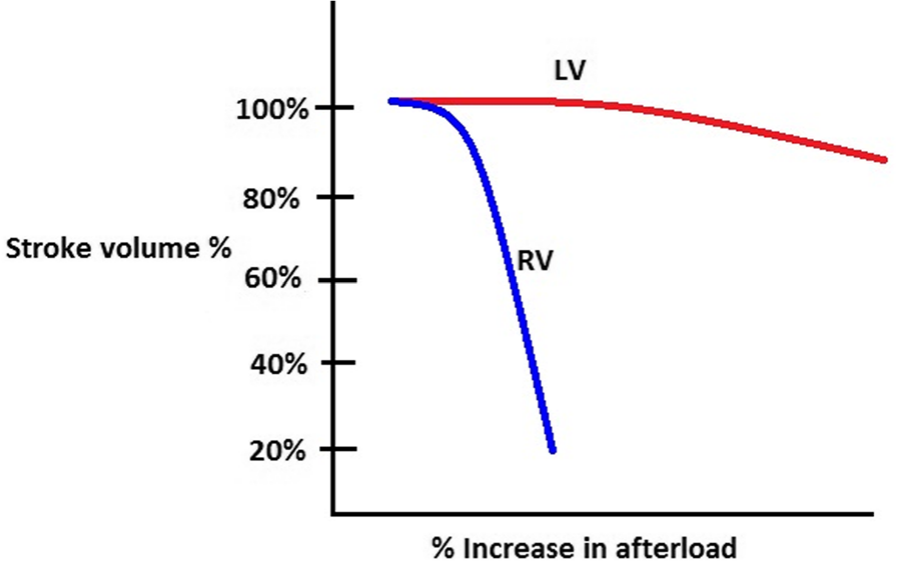
Relationship of afterload and stroke volume. Right ventricular (red) stroke volume response to increases in afterload is devastating compared to the left ventricular (blue) stroke volume response which is more gradual

**Fig. 4:**
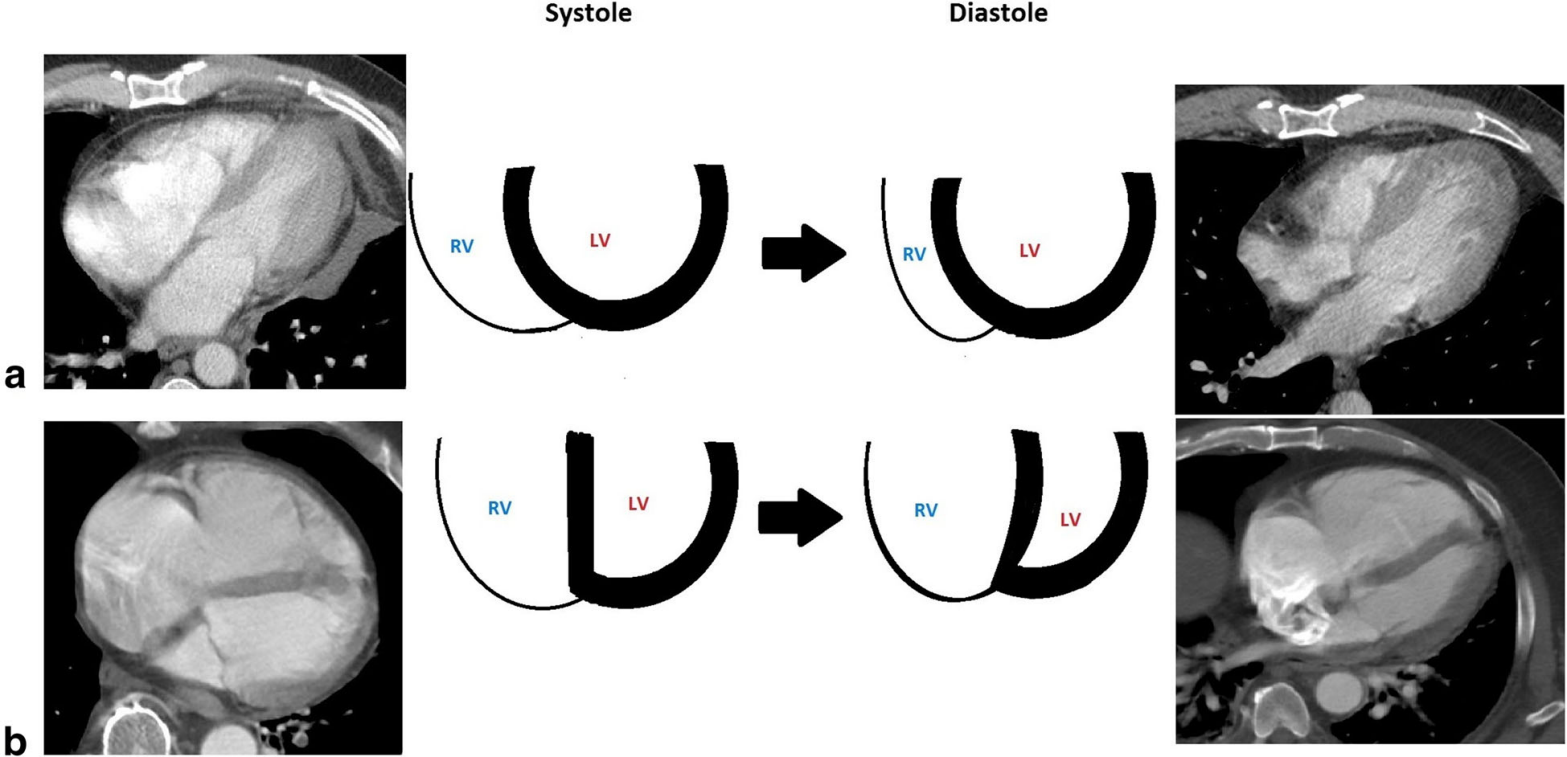
Normal and abnormal appearance of right and left ventricles due to afterload. **a** Depicts normal systolic and diastolic appearance of the right and left ventricles. **b** Demonstrates a dilated right ventricle that impinges on the left ventricle due to increased afterload

**Fig. 5:**
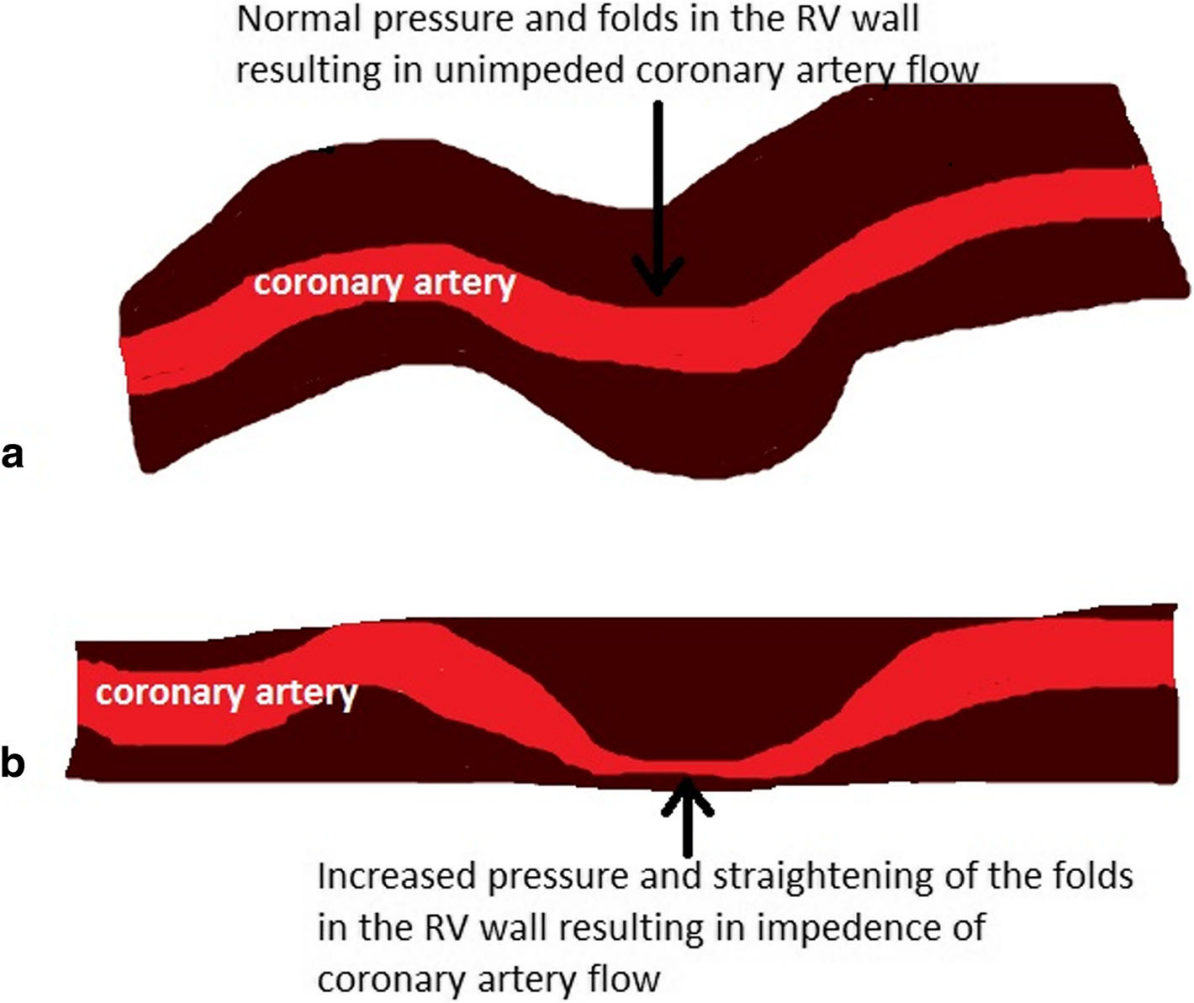
Pictorial depiction of the compromise of the coronary arteries. **a** Depicts uninhibited flow of blood through a coronary artery as it passes through the folds (arrow) of the right ventricular wall. **b** Depicts a coronary artery with region of stenosis due to straightening of the folds in a dilated RV wall

**Fig. 6:**
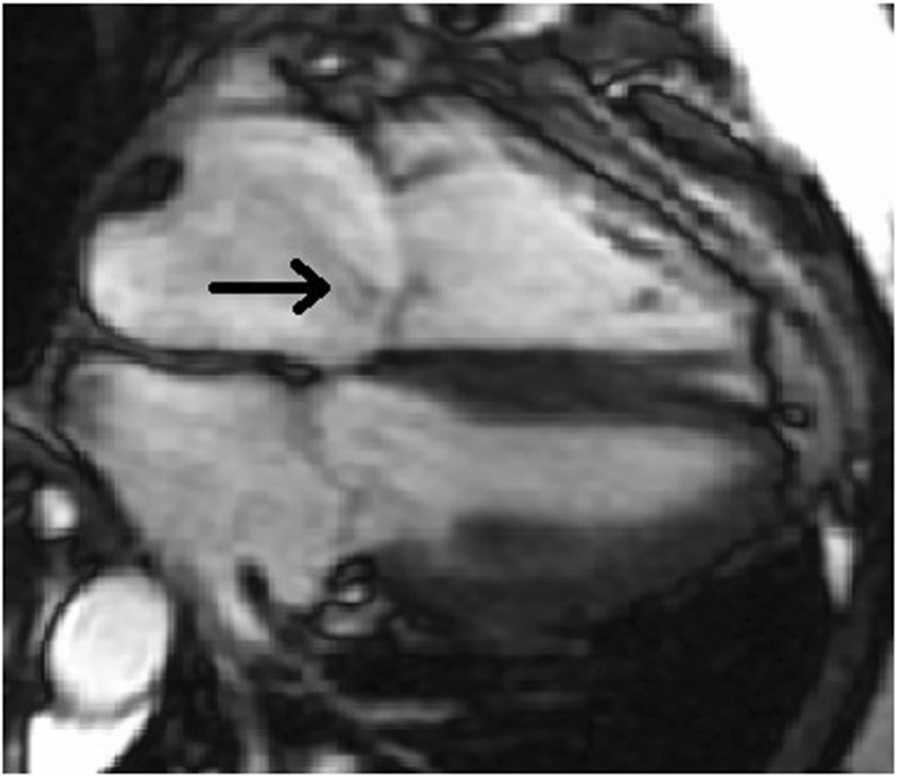
Tricuspid regurgitation in a dilated right ventricle and right atrium. Fiesta cine sequence MRI image demonstrates a four-chamber view with incomplete closure of the tricuspid valve due to right ventricular dilatation resulting in tricuspid valve regurgitation (arrow)

Because the right ventricle is thin-walled and compliant, it is ill-equipped for acute increases in afterload compared to the muscular thick-walled noncompliant left ventricle. When there are acute incremental increases in afterload, there are drastic decreases in stroke volume, or forward flow of blood (Fig. 3) [6, 7]. With acute increases in afterload, the compliant right ventricle cannot unload sufficiently and dilates which gives rise to three important outcomes: (1) the dilated RV pushes the interventricular septum toward the left ventricle, impinging on the left ventricle, resulting in underfilling of the left ventricle (Fig. 4), decreased left ventricle output, and therefore decreased supply to the coronary arteries; (2) right ventricular intramuscular pressure increases, straightening normal muscular folds, impeding coronary blood flow in the right heart wall (Fig. 5), further leading to ischemia; and (3) stretching of the annulus of the tricuspid valve results in tricuspid regurgitation (Fig. 6), further decreasing forward flow of blood from the right ventricle [5, 8–10].

## Acute pulmonary embolus and right ventricular compromise

RV afterload increases with pulmonary emboli. In patients without pre-existing cardiopulmonary disease, 25–30% of the pulmonary vasculature must be occluded before the pulmonary artery pressure rises, increasing the RV afterload [11]. The RV compensates until greater than 50–75% of the pulmonary vasculature is obstructed by emboli with a pulmonary artery pressure increase above 40 mmHg [11]. Afterload is further worsened when hypoxia, induced by the emboli, causes localized vasoconstriction by stimulating the release of vasoactive mediators, such as serotonin, thromboxane, and histamine [9]. When afterload has reached the critical level, the RV dilates, the LV underfills, and decreases supply to the coronary arteries. Perfusion to the right ventricle drops because there is decreased output to the coronary arteries and increased intramuscular pressure impeding the coronary artery flow, leading to right ventricular ischemia [12].

**Fig. 7:**
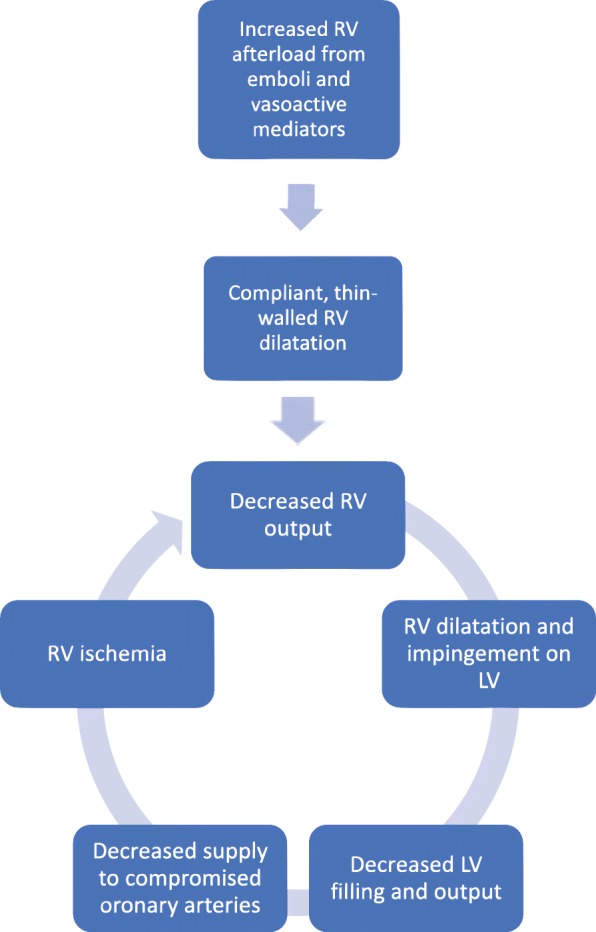
Hemodynamic collapse in PE. Diagram demonstrates a spiral resulting in hemodynamic collapse in patients with massive and submassive PE

**Fig. 8:**
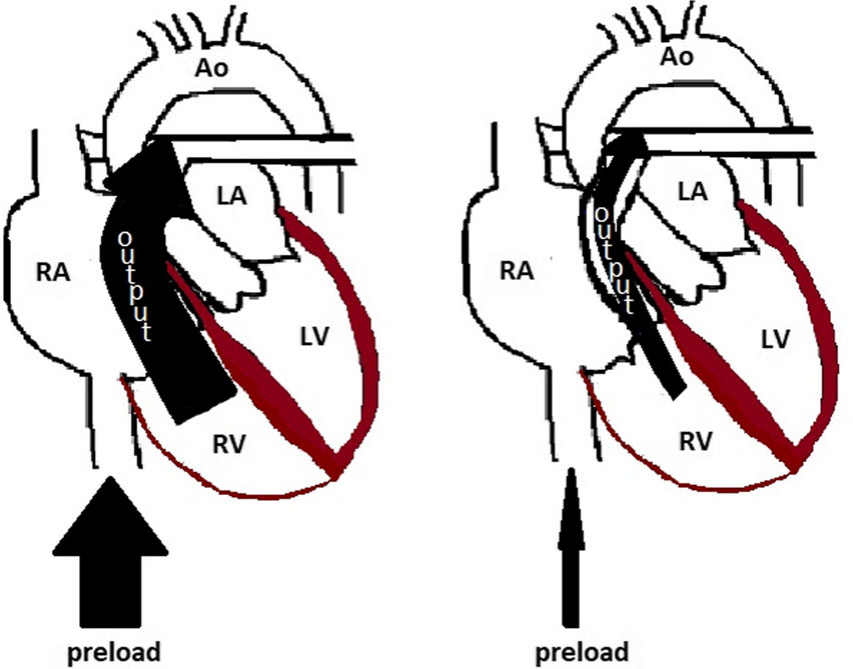
Depiction of the effects of preload on output from the RV to the pulmonary arteries. If preload is decreased due to venous dilation and pooling of the blood in the dilated venous system, there is decreased filling of the RV and decreased available volume for output

As the right ventricle becomes ischemic, its contractility further suffers, further decreasing right ventricular output, increasing right ventricular dilatation, and decreasing left ventricular output, resulting in a downward hemodynamic spiral that augments itself and leads to cardiogenic shock (Fig. 7) [8, 13]. It should be noted that medications such as propofol used for the induction and maintenance of general anesthesia decrease venous return to the right heart, or the preload, due to peripheral venous dilatation, further compromising the RV’s output and ability to perform against an elevated afterload (Fig. 8) [14].

## Differences in massive and submassive PE

Massive or high-risk and submassive or intermediate-risk PE are divided by hemodynamic presentation (Table 1) [15]. Massive PE occurs in about 5% of patients with PE and carries a mortality rate of 18–65% [2]. It is defined as PE with sustained hypotension (systolic BP < 90 for at least 15 min), the need for inotropic support, or persistent bradycardia (HR < 40 bpm with signs or symptoms of shock) [15]. Persistent bradycardia may be a result of complete atrioventricular (AV) block due to injury to the right bundle branch that runs superficially in the right ventricular wall and in the intraventricular septum, and is sensitive to acute right ventricular dilatation, in a patient with pre-existing electrical conduction system disease like left bundle branch block (LBBB) [16]. Of note, the right bundle branch can also be disturbed during pulmonary catheterization, a technique that may be used by interventional radiologists in diagnosing and treating massive and submassive PE, causing a complete AV block in a patient with existing LBBB [17]. Therefore, a temporary pacemaker placement should be considered in patients with LBBB [17].

**Table 1:** PE subtypes, % of patients, clinical definition, and mortality rate

PE subtypes	Massive PE	Submassive PE	Simple PE
% of PE patients	≈ 5%	≈ 40%	≈ 55%
Clinical definition	Sustained hypotension (systolic < 90 mmHg for at least 15 min), need for inotropic support, persistent profound bradycardia (HR < 40 bpm with signs or symptoms of shock)	Systemically normotensive (systolic BP > 90 mmHg), myocardial ischemia (elevated troponins, ECG changes), and/or RV dysfunction (dysmotility on Echo, Increased RV/LV ratio > 0.9, elevated BNP/pro BNP), ECG changes)	Systemically normotensive (systolic BP > 90 mmHg), no RV dysfunction, no myocardial ischemia
Mortality	18–65%	5–25%	Up to 1%

Submassive PE, seen in about 40% of patients with PE, carries a 5–25% mortality rate (Table 1) [2]. Patients with submassive PE are systemically normotensive with evidence of myocardial ischemia or dysfunction as demonstrated by elevated troponins and electrocardiogram (ECG) changes, and/or RV dysfunction demonstrated by decreased motility on echo, increased right ventricle/left ventricle (RV/LV) ratio greater than 0.9 on Echo or CT, elevation of beta natriuretic peptide (BNP) and pro-BNP which mark heart failure, and ECG changes [15].

The remaining 55% of patients with PE present with nonmassive or low-risk PE, also called uncomplicated PE, with a mortality rate of up to 1% (Table 1) [2]. Patients with simple PE are systemically normotensive, without right ventricular dysfunction or myocardial ischemia.

## Clinical considerations in acute PE and RV compromise

**Fig. 9:**
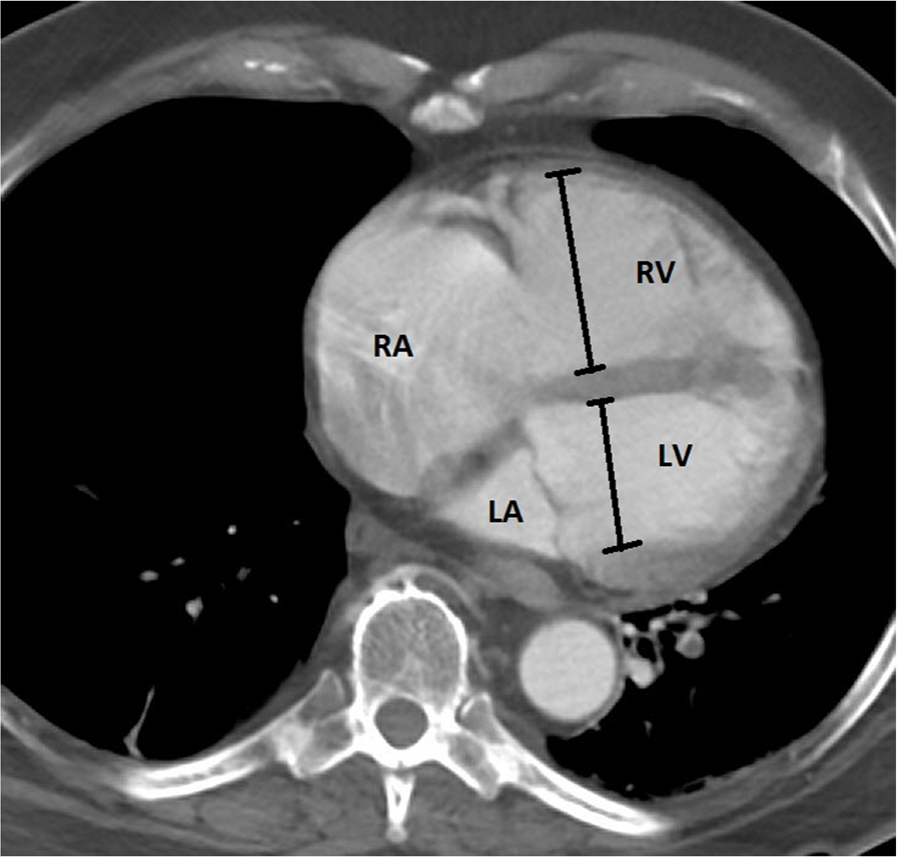
Measurement of RV/LV ratio. Axial CT images demonstrate the best approximated four-chamber view. Calipers are placed from inner wall to inner wall at the maximal diameter on a view that best approximates the four-chamber view

When evaluating a CT in a patient with PE to assess for RV compromise and significance of PE burden, one may note the RV/LV ratio which has been associated with clinical outcome [18–20]. The RV/LV ratio is determined by measuring the maximal RV and LV diameters from inner wall to inner wall on the axial slice that best approximates the four-chamber view (Fig. 9) [21]. A value > 0.9 is considered abnormal. It is important to note that an axial RV/LV diameter ratio is no less accurate than a reformatted four-chamber RV/LV diameter ratio [21].

**Fig. 10:**
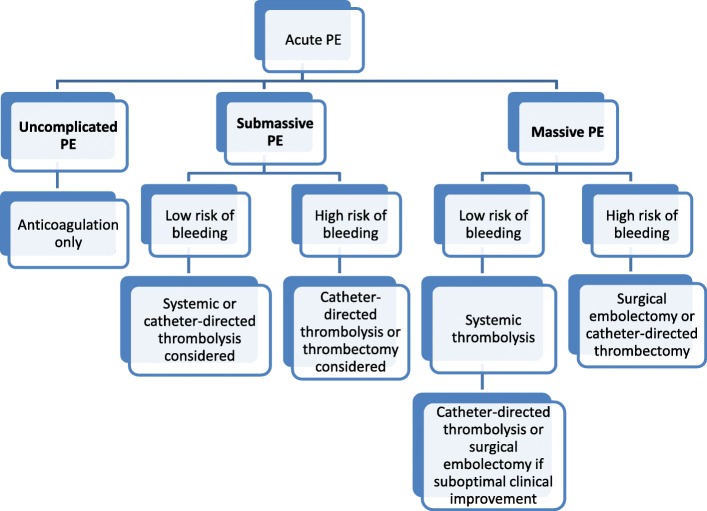
Treatment algorithm for acute PE. Diagram demonstrates an algorithm for treatment of acute PE

**Fig. 11:**
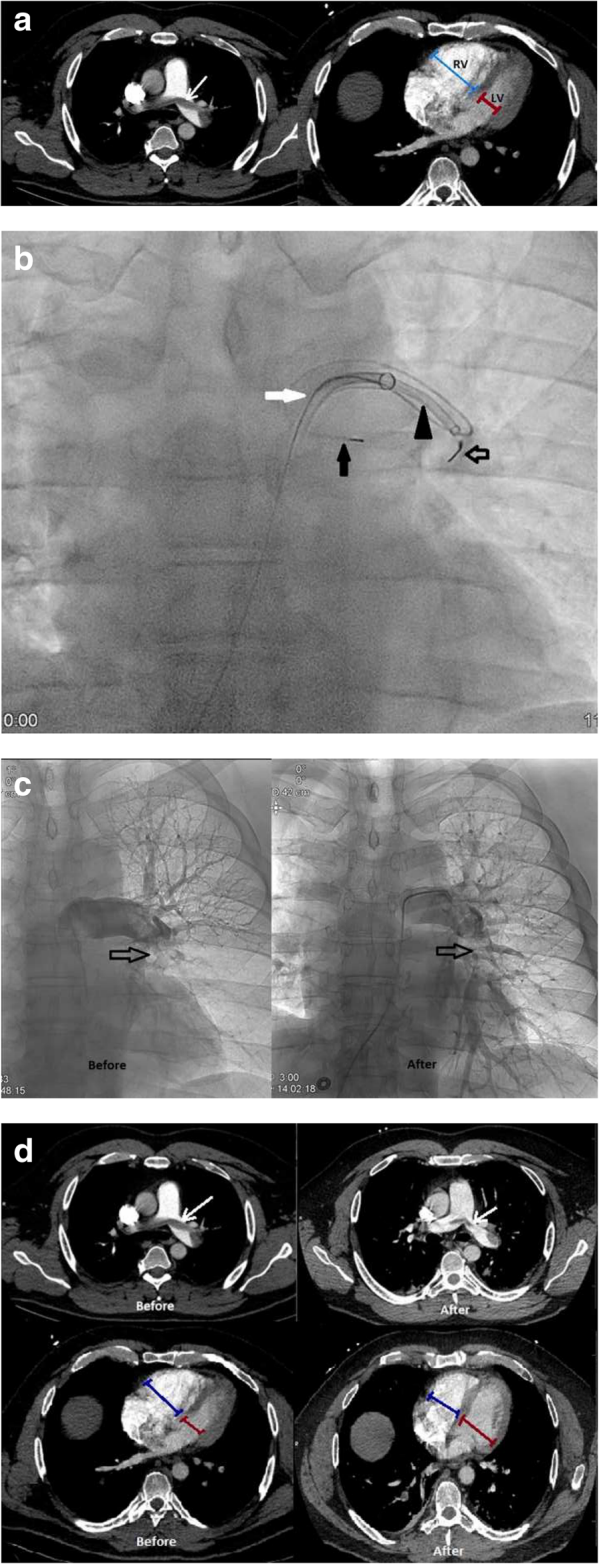
A 44-year-old male with a left lower extremity DVT after trauma presenting with massive PE, 8 days after initiation of anticoagulation for the DVT. Patient was hypotensive with a BP of 78/45 and troponin I elevation of 0.83 ng/mL. **a** CTA demonstrates a saddle pulmonary embolus (white arrow) and increased RV/LV ratio with blue calipers demonstrating the diameter of the RV and the red calipers demonstrating the diameter of the LV. **b** Fluoroscopic images of aspiration thrombectomy. A 90 cm 8Fr Sheath (white arrow) through which is passed an Indigo CAT-8 thrombectomy device (black arrowhead, Indigo, Penumbra, Alameda, CA, USA). Black closed arrow demonstrates a safety wire to stabilize access to the left pulmonary artery. Black open arrow demonstrates the Indigo separator to clear thrombus from the opening of the catheter. **c** Pulmonary angiogram demonstrates before and after catheter-directed therapy pictures. Arrow demonstrates improved perfusion to the lower lobe. **d** CTA demonstrates before and after catheter-directed therapy images with improvement in thrombus burden and before and after catheter-directed therapy images with improvement in RV/LV ratio

**Fig. 12:**
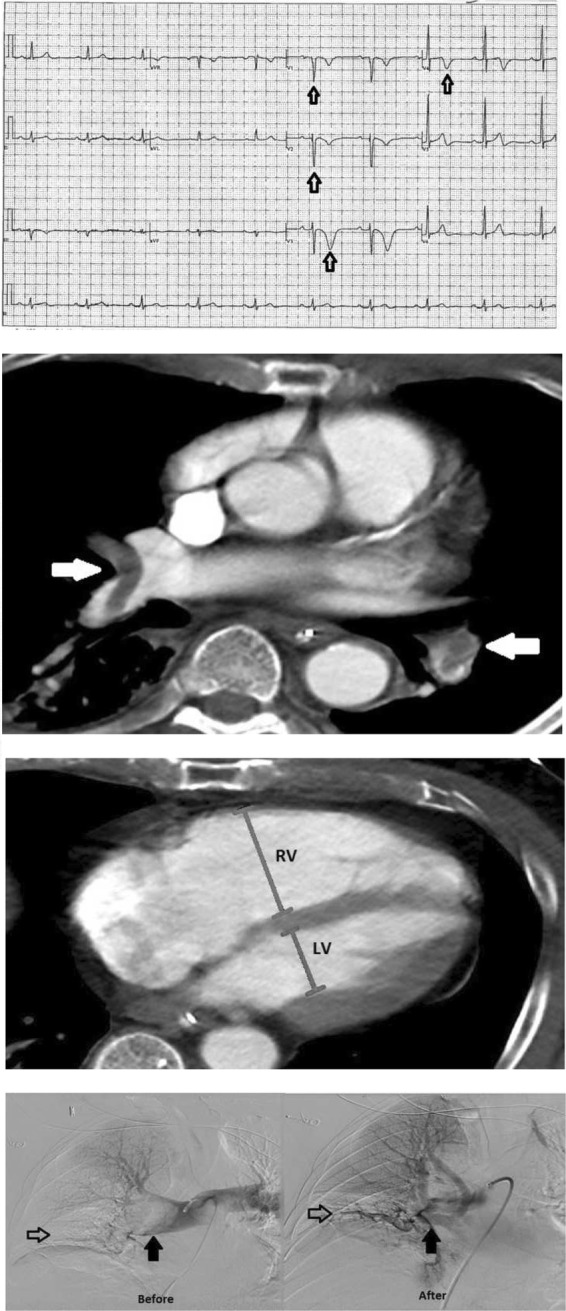
A 63-year-old man with renal cell carcinoma and hemorrhagic brain metastases presenting with submassive PE, felt not safe for anticoagulation. The patient was normotensive but had decreased RV motility on Echocardiogram, pro-BNP of 930 pg/mL, and troponin I elevation of 0.46 ng/mL, and ECG changes. **a** ECG in a patient with submassive PE depicts ischemia demonstrates inverted T waves in V1–V4 leads, a representative ECG abnormality that may be seen with submassive PE. **b** CTA demonstrates right main pulmonary artery and lobar pulmonary emboli (white arrows). **c** CTA demonstrates increased RV/LV ratio with blue calipers measuring the RV and red calipers measuring the left ventricle. **d** Before and after mechanical thrombectomy pulmonary angiogram images depicting improved patency of the right main pulmonary artery (solid arrow) and perfusion to the right lower lobe (open arrow) compared to before

Treatment of massive PE is aimed at relieving the RV afterload to improve RV function. Treatment methods include pulmonary artery endarterectomy, systemic IV thrombolytics, and relatively more recent, percutaneous intervention such as catheter-directed thrombectomy and thrombolysis performed by interventional radiologists (Figs. 10 and 11) [15]. Historically, treatment of submassive PE has been only anticoagulation. However, given the compromise of the RV, other treatments such systemic low-dose IV thrombolytics and percutaneous interventions like catheter-directed thrombectomy and thrombolysis may be performed by interventional radiologists (Figs. 10 and 12) [15]. For patients with uncomplicated PE, treatment remains anticoagulation only (Fig. 10) [15]. Suboptimal or incomplete treatment of any PE can sometimes result in CTEPH [3]. Figure 10 provides a treatment algorithm for acute PE [15].

## Chronic thromboembolic pulmonary hypertension

CTEPH is a chronic progressive pulmonary vascular complication of acute PE severely and progressively affecting the RV, that occurs in 1.5 to 3.8% of patients with one or more episodes of acute PE [3, 4]. It is a subtype of pulmonary hypertension characterized by a mean pulmonary artery pressure ≥ 25 mmHg due to obstructive fibrotic thromboembolic material in the pulmonary arteries from remodeled unresolved pulmonary embolus [3]. Patients with infected ventriculoatrial shunts for the treatment of hydrocephalus, indwelling catheters and leads, thyroid replacement therapy, malignancy, and chronic inflammatory disorders, such as osteomyelitis and inflammatory bowel diseases, have a higher risk of developing CTEPH [22]. Studies have demonstrated an association with inflammatory markers such as C-reactive protein (CRP), IL-10, monocyte chemotactic protein-1, macrophage inflammatory protein-1α, matrix metalloproteinase (MMP)-9, interferon-γ-induced protein (IP)-10, and tumor necrosis factor-α in these patients [23–25]. In addition, the bacterium *Staphylococcus aureus* has been harvested in the blood and thrombi of some of these patients [22, 26]. Therefore, it is believed that inflammation and infection play a part in the development of CTEPH [27]. The process involves progressive remodeling of residual thrombi into a fibrotic material of collagen, elastin, inflammatory cells, re-canalized vessels, and rarely calcification, progressively obstructing the pulmonary vasculature in the form of bands, webs, stenoses, and occlusions resulting in a chronically increased afterload for an ill-equipped right ventricle [27].

## CTEPH and right ventricular compromise

**Fig. 13:**
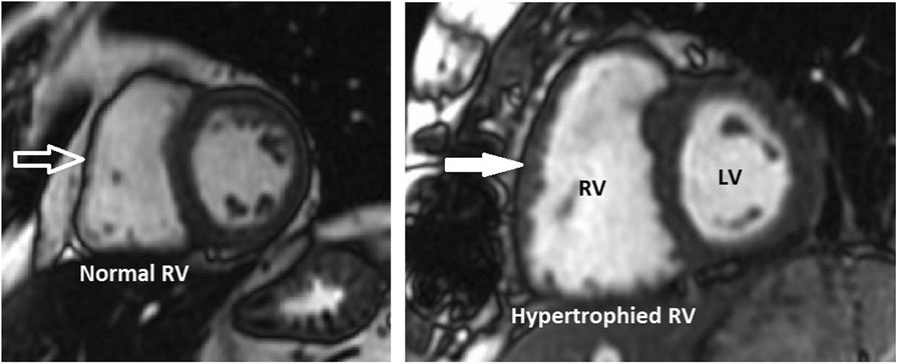
Right ventricular wall hypertrophy. Short axis SSFP MRI images demonstrate normal (open white arrow) and hypertrophied (closed white arrow) RV wall

**Fig. 14:**
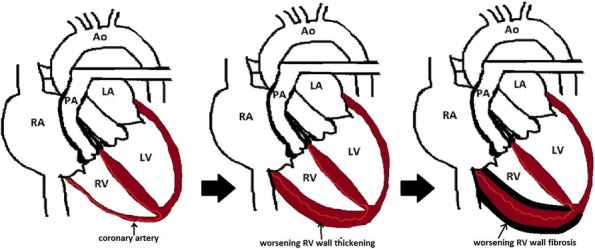
Diagram demonstrates adaptive and maladaptive RV wall thickening. The RV wall progressively thickens then becomes necrotic and fibrotic as coronary arteries cannot meet the demands of the RV wall

**Fig. 15:**
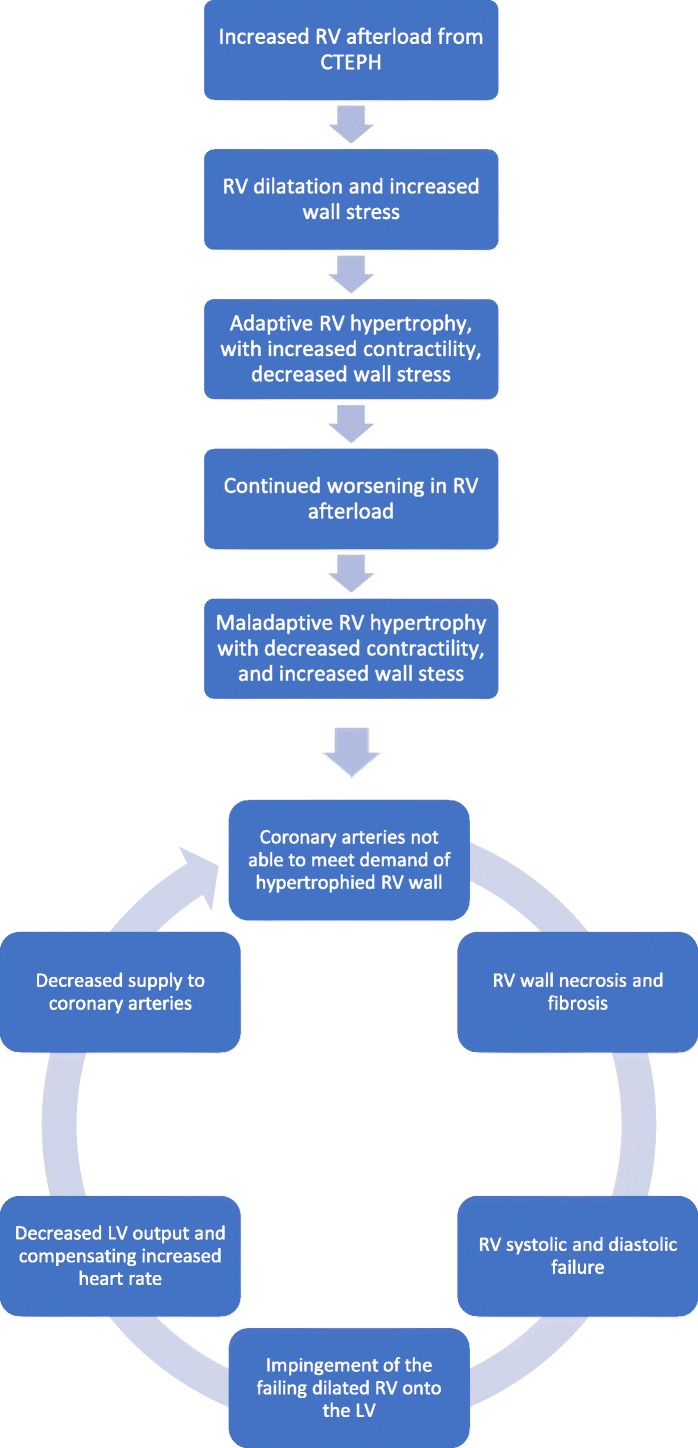
The events of CTEPH and progressive right heart failure. Diagram demonstrates the events leading to the spiral in CTEPH that leads to right heart failure and ultimately death if no effective intervention is performed

In CTEPH, to accommodate the RV afterload and wall stress to the RV, adaptive remodeling occurs with RV wall hypertrophy (Fig. 13), through the addition of sarcomeres, the functional unit of striated muscle, in a process called adaptive hypertrophy [28, 29]. Adaptive right ventricular wall hypertrophy results in decreased wall stress and improved pumping capability, with RV function more closely mimicking that of the LV [30]. However, the ill-equipped RV is not capable of sustaining the long-term progressively increased afterload and remodeling becomes maladaptive [29]. RV dilatation and wall hypertrophy increase oxygen demand to a level which the coronary artery blood flow cannot meet, resulting in ischemia, necrosis, and fibrosis of the RV wall (Fig. 14), worsening contractility of the right ventricle, and right ventricular failure. Right ventricular failure leads to further RV dilatation impinging on the LV, worsening LV filling, decreased LV stroke volume, increased heart rate to compensate for the decrease in the LV stroke volume (SV), and decreased output to the coronary arteries [28, 29]. Decreased output to the coronary arteries therefore occurs by both (1) decrease in the LV SV and (2) increase in the heart rate to compensate for the decreased in the SV [28, 29]. The increase in heart rate shortens the time coronary artery blood can flow, which occurs during diastole [31]. The compromise of the coronary arteries is accentuated as the coronary arteries already cannot meet the demand of the hypertrophied dilated RV [31]. Moreover, in addition to RV systolic dysfunction, there is RV diastolic dysfunction, as the hypertrophied, noncompliant, stiff, fibrotic RV wall, requires more time to relax than normal and cannot fill as normal in its allotted time [32, 33]. As with acute RV dysfunction, the chronic RV dysfunction creates a progressive downward spiral leading to severe heart failure (Fig. 15).

## Clinical considerations in CTEPH

CTEPH may occur several months or years after the acute PE event, which may be silent. Patients must receive at least 3 months of effective anticoagulation treatment with an acute PE before diagnosis of CTEPH [34]. Symptoms of CTEPH are indicative of RV failure and include new or ongoing worsening shortness of breath, dyspnea on exertion, inability to tolerate activity, and less often hemoptysis and should prompt further workup with imaging [34].

**Fig. 16:**
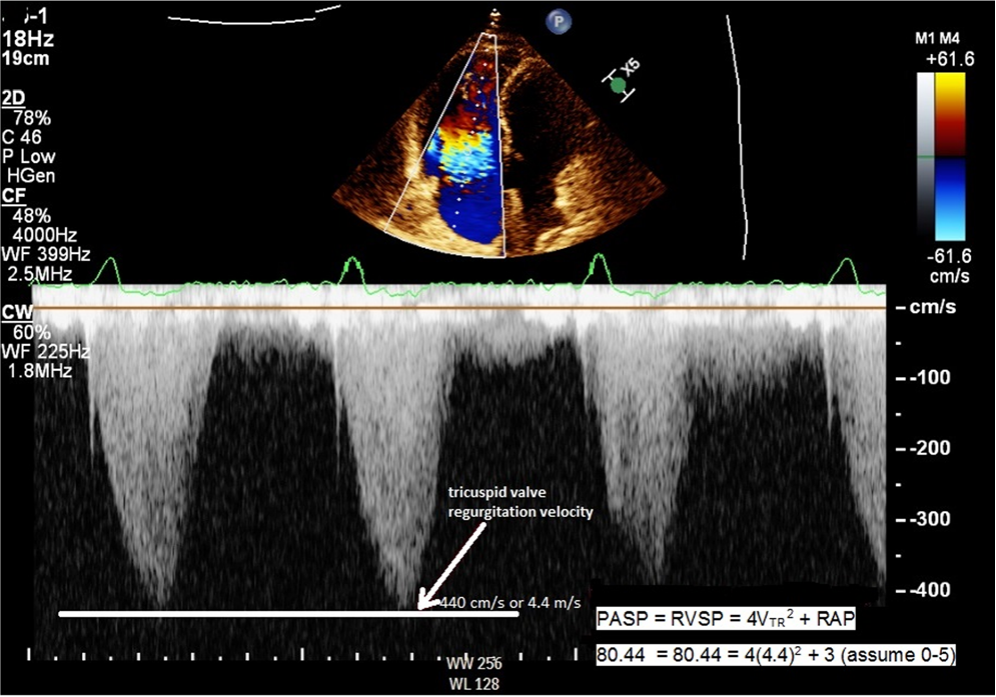
Pulmonary artery systolic pressure (PASP) calculated from a tricuspid jet on echocardiogram. Estimation of PASP utilizing the tricuspid regurgitation jet velocity can be performed according the depicted equation where RVSP is right ventricle systolic pressure and RAP is right atrial pressure which is the same as Jugular venous pressure

**Fig. 17:**
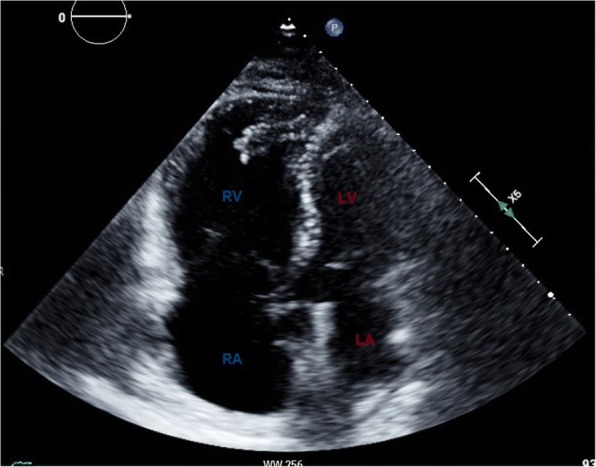
Four-chamber view on echocardiogram in a patient with CTEPH. Echo demonstrates RV and RA dilatation compared to the LV and LA (labeled)

**Fig. 18:**
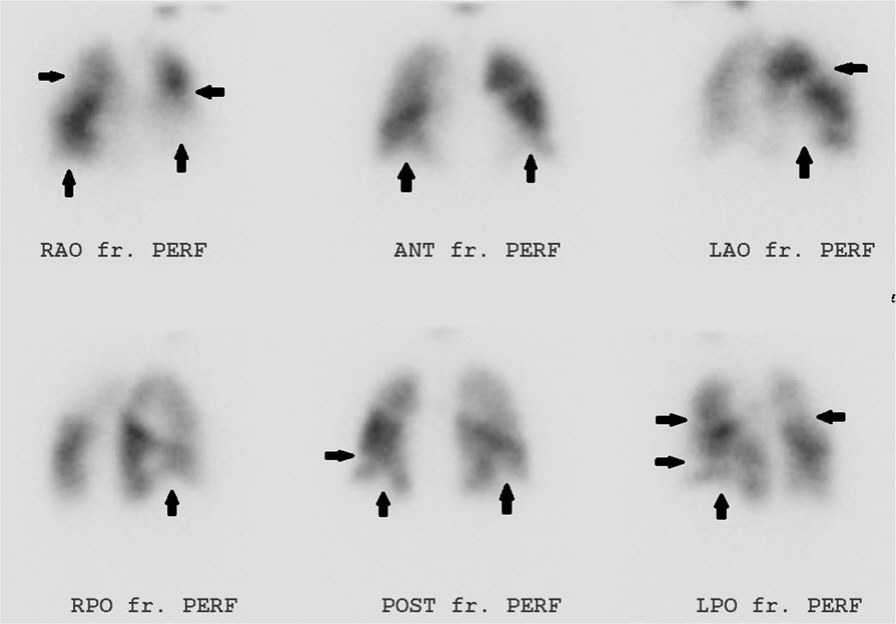
Perfusion images of a V/Q scan in a patient with CTEPH. Multiple perfusion segmental defects in a patient with normal ventilation (arrows)

**Fig. 19:**
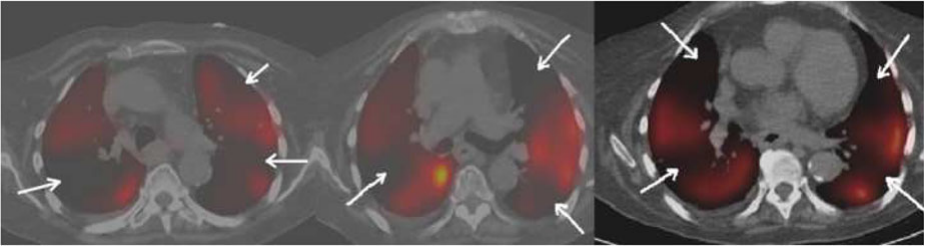
SPECT perfusion scan in a patient with CTEPH. SPECT demonstrates multiple perfusion defects (arrows)

**Fig. 20:**
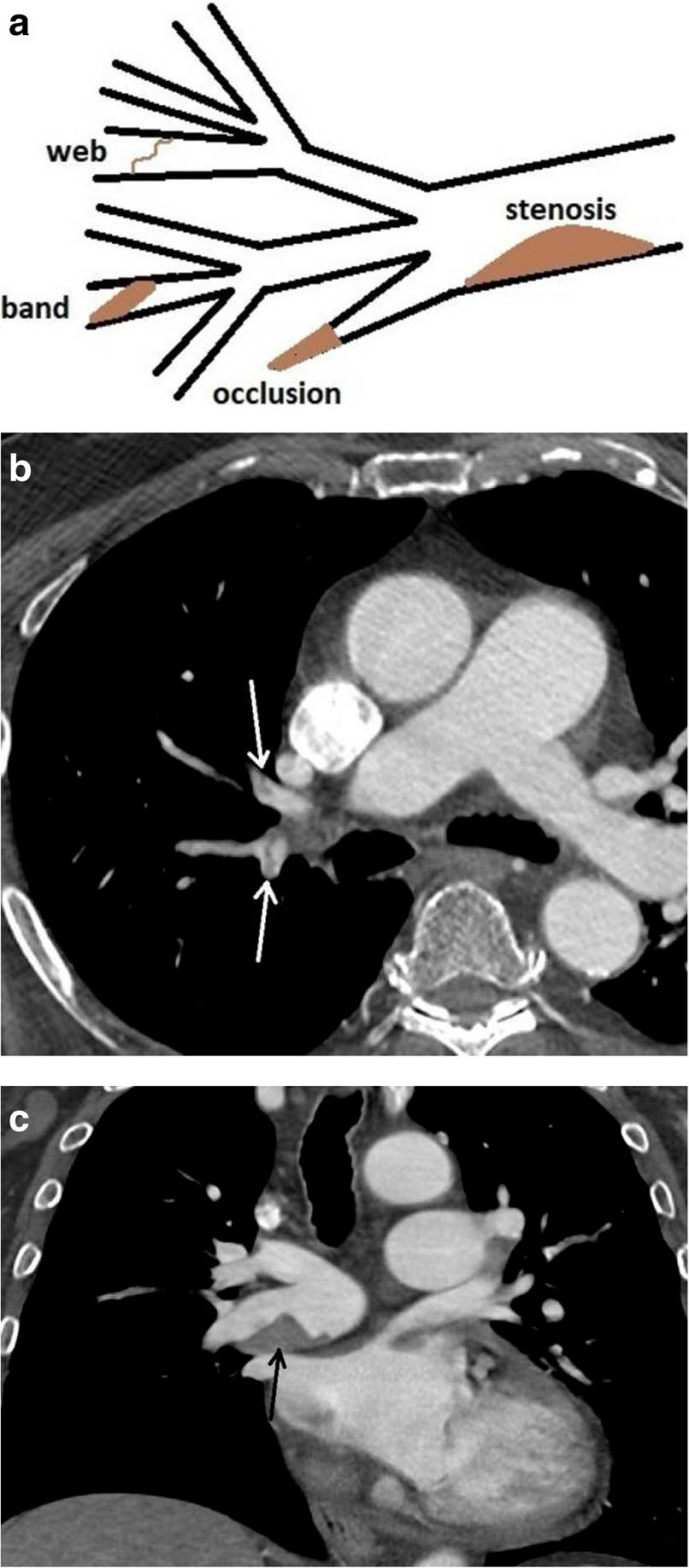
Configurations in CTEPH. **a** Figure demonstrating findings of CTEPH including stenosis, web, band, and occlusion. **b** CTA axial image of web/band (white arrows). **c** CTA axial image demonstrates eccentric thrombus narrowing the lumen of the right main pulmonary artery (black arrow)

**Fig. 21:**
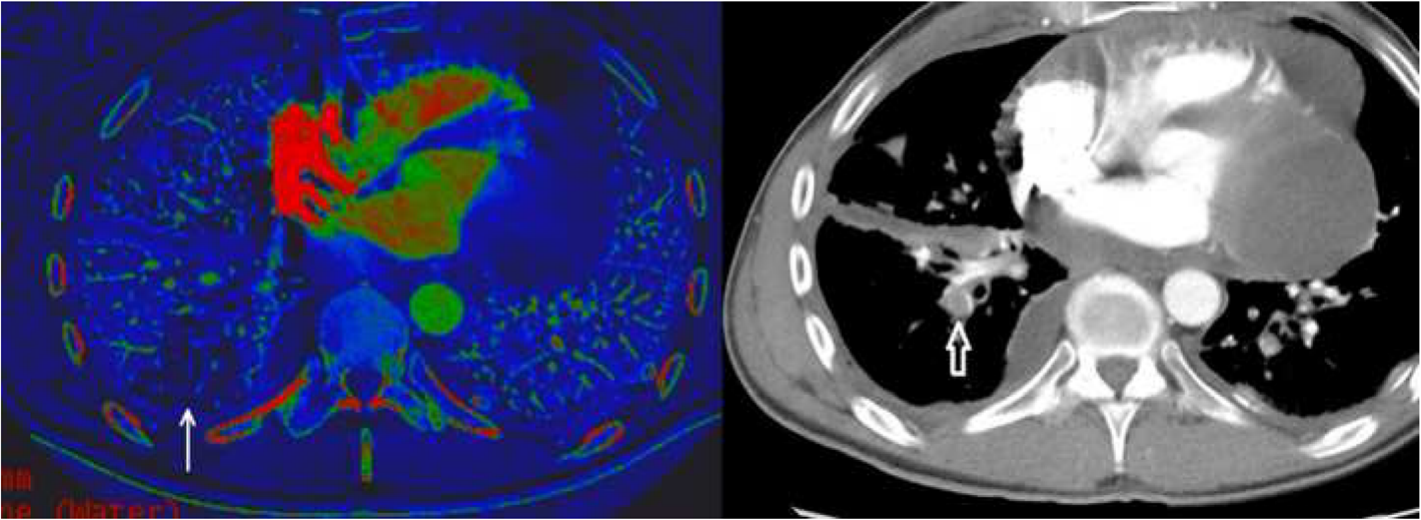
Dual energy CT in a patient with pericardial and pleural metastases, with a PE. Iodine map is shown with perfusion defect (white arrow). This is correlated to the region with the PE in the right lower lobe (open white arrow)

**Fig. 22:**
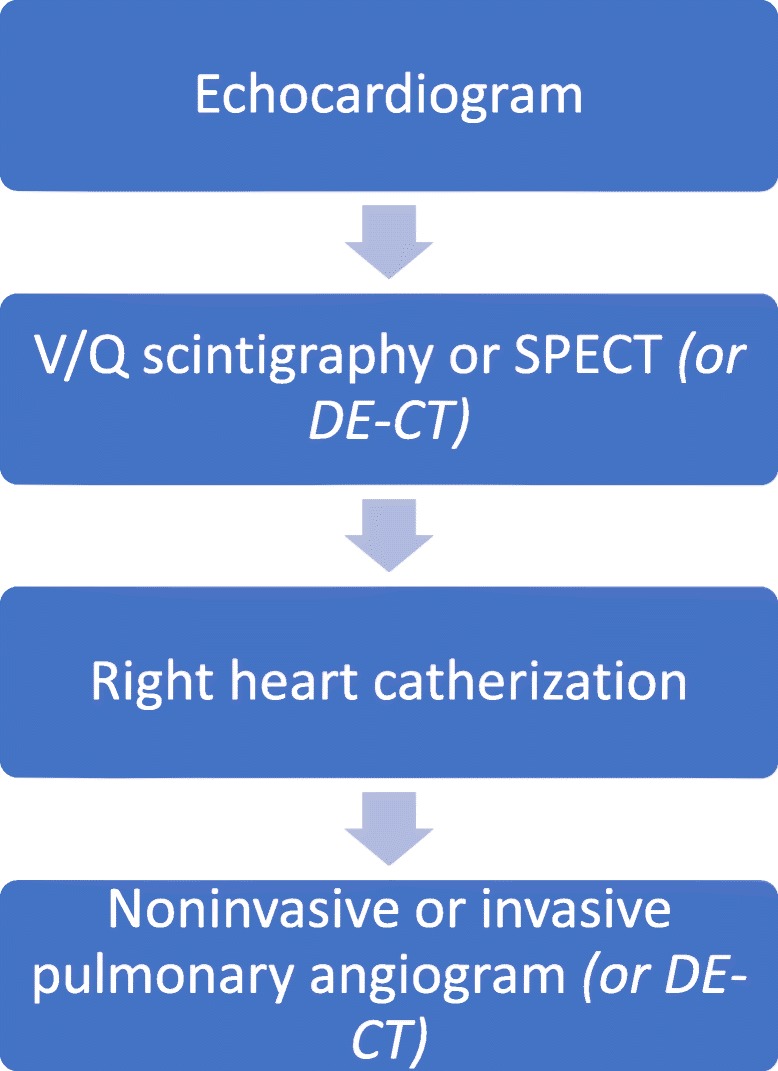
Imaging algorithm in CTEPH. Diagram demonstrates an algorithm for the imaging in patients with suspected CTEPH

Imaging reflects the consequences of CTEPH on the pulmonary vasculature and/or the right ventricle. Imaging often begins with an echocardiogram in patients with suspected CTEPH, where the pulmonary artery systolic pressure (PASP), estimated by using the velocity of the tricuspid regurgitation jet (Fig. 16), may be elevated. Echocardiography may also demonstrate the deterioration of the right heart with the right ventricular size increased and motility compromised (Fig. 17) [35]. Additionally, patients with suspected CTEPH may undergo planar (Fig. 18) or single-photon emission computed tomography (SPECT) ventilation/perfusion (V/Q) scan (Fig. 19), which remains the screening imaging tool of choice where one notes mismatched segmental defects [36]. After echocardiogram and V/Q scan, pulmonary vasculature hemodynamics are confirmed with a right heart catheterization (RCH) where pulmonary artery pressure (PAP) is ≥ 25 mmHg, pulmonary capillary wedge pressure (PCWP) ≤ 15 mmHg, and pulmonary vascular resistance (PVR) > 240 dynes s^−1^ cm^−5^ is noted in CTEPH [37]. RCH is very important pre-treatment, as patients with a preoperative PVR > 1000 dynes s^−1^ cm^−5^ have a significantly higher mortality rate than those with a preoperative PVR < 1000 dynes s^−1^ cm^−5^ [38]. Finally, in CTEPH, invasive pulmonary angiogram and CT pulmonary angiograms will demonstrate the bands, webs, stenoses, and occlusions obstructing the pulmonary vasculature (Fig. 20a–d) resulting in the progressive severe RV failure, and can be used to plan treatment [27]. Dual energy computed tomography can be utilized to combine the benefits of a V/Q scan demonstrating perfusion defects and a pulmonary angiogram demonstrating the abnormality of the pulmonary vasculature (Fig. 21) [39]. Table 2 provides a summary of imaging findings in CTEPH and Fig. 22 provides an imaging algorithm for suspected CTEPH [34].

**Table 2:** Imaging findings in CTEPH. (PAP = pulmonary artery pressure, PCWP = pulmonary capillary wedge pressure)

Modality	Findings
Echocardiogram	• PAP > 25 mmHg • Right atrial and right ventricular dilatation • Reduced right ventricular contractility
Nuclear medicine studies	• Segmental wedge-shaped mismatched defects on perfusion scan
Right heart catheterization	• PAP is ≥ 25 mmHg • Pulmonary capillary wedge pressure (PCWP) ≤ 15 mmHg • Pulmonary vascular resistance is > 240 dyn-sec-cm-5
Invasive and noninvasive pulmonary angiogram	Invasive: • Stenoses and occlusions Noninvasive: • Bands, webs, stenoses, and occlusions
Dual energy CT	CTA portion of study: • Bands, webs, stenoses, and occlusions Perfusion blood volume of study • Decreased perfusion in regions of involvement

**Fig. 23:**
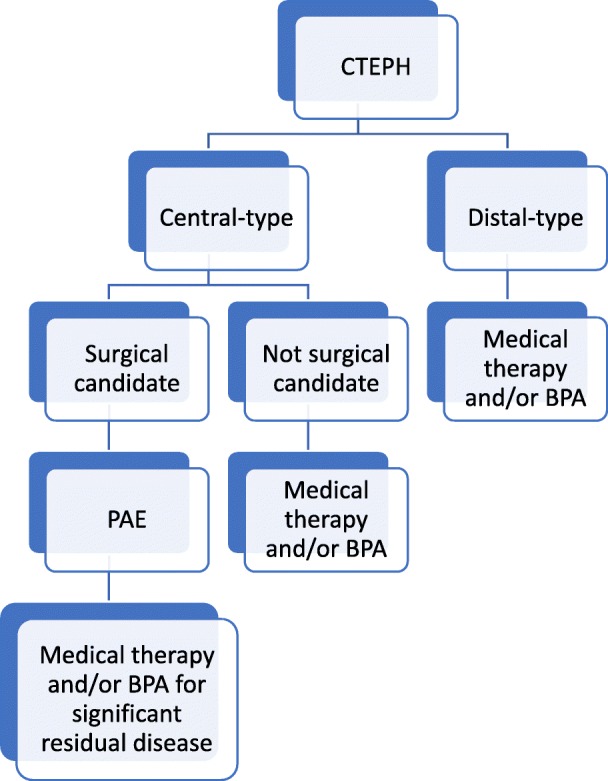
Treatment algorithm of CTEPH. Diagram demonstrates an algorithm for the treatment of central-type and distal-type CTEPH

**Fig. 24:**
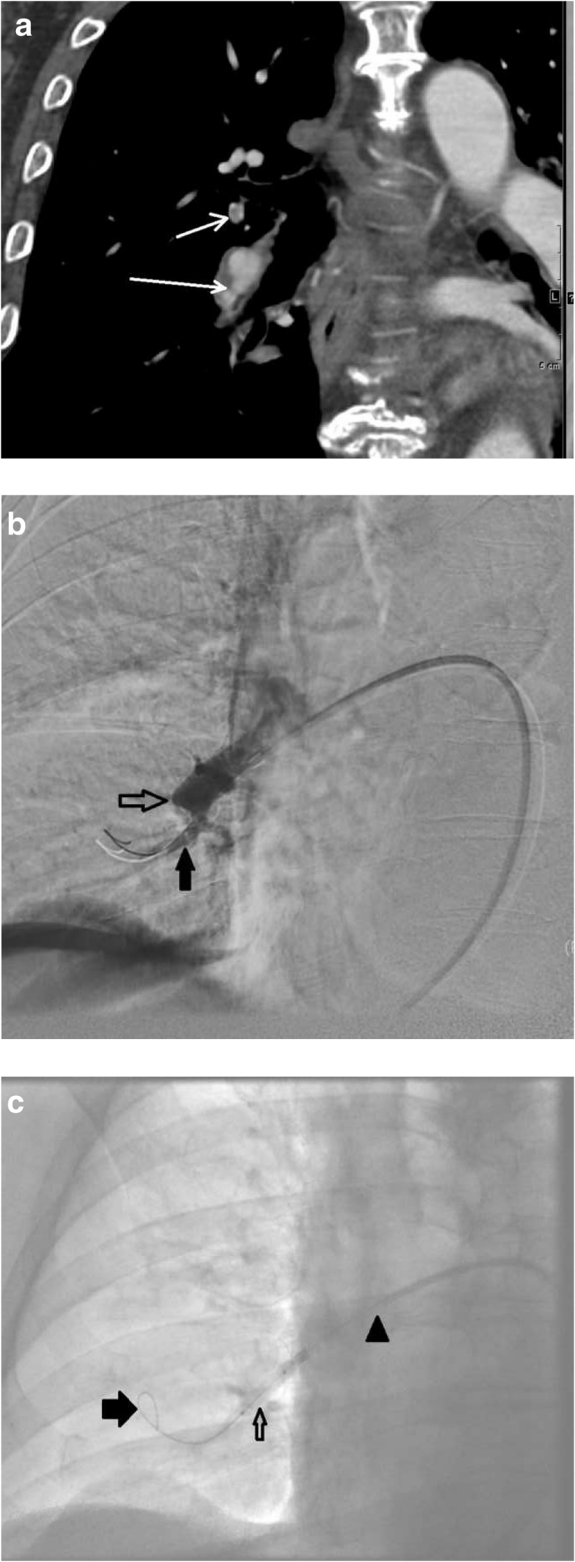
Imaging and treatment in a patient with CTEPH. An 82-year-old male patient, nonsurgical candidate, with central-type and distal-type CTEPH. **a** CTA demonstrating webs and bands (white arrows). **b** Region of stenosis (solid black arrow) and occlusion (open black arrow) due to webs and bands. **c** Fluoroscopic image of balloon pulmonary angioplasty. A 4 mm balloon (open black arrow), passed through a 7 Fr 90 cm Brite Tip sheath (Cordis, Milpitas, CA, USA, arrow head), over a V18 (Boston Scientific, Malborough, MA) working wire (solid black arrow). **d** Pulmonary angiogram demonstrates revascularized segmental pulmonary arteries (black arrows)

Treatment for CTEPH is aimed at relieving the afterload for the deteriorating RV (Fig. 23). Pulmonary artery endarterectomy is utilized for patients with more central disease (central-type CTEPH) and patients who are surgical candidates [40]. For more distal disease in the segmental and subsegmental regions (distal-type CTEPH), medical therapy with Riociguat and more recently balloon pulmonary angioplasty (BPA) may be performed [40–42]. BPA is a revolutionary procedure performed by an interventional radiologist that changes the face of inoperable and distal-type CTEPH by using angioplasty balloons to disrupt regions of obstruction in the pulmonary vasculature and decrease RV afterload (Fig. 24) [42]. Figure 23 provides a treatment algorithm for CTEPH [34].

## Conclusion

Pulmonary embolus can be devastating leading to acute and chronic RV failure. Understanding the pathophysiology of RV failure in massive and submassive PE and CTEPH is the key factor in the justification for percutaneous interventions in selected patients. In addition, it aids communication with patients, their families, and the referring clinicians.

## Abbreviations

AV: Atrioventricular
BNP: Beta natriuretic peptide
BP: Blood pressure
BPA: Balloon pulmonary angioplasty
CRP: C-reactive protein
CTEPH: Chronic thromboembolic pulmonary hypertension
ECG: Electrocardiogram
HR: Heart rate
IL: Interleukin
IP: Induced protein
LBBB: Left bundle branch block
LV: Left ventricle or left ventricular
MMP: Metalloproteinase
PAP: Pulmonary artery pressure
PASP: Pulmonary artery systolic pressure
PCWP: Pulmonary capillary wedge pressure
PE: Pulmonary embolus
PVR: Pulmonary vascular resistance
RCH: Right heart catheterization
RV: Right ventricle or right ventricular
RV/LV: Right ventricle/left ventricle
SPECT: Single-photon emission computed tomography
SV: Stroke volume
V/Q: Ventilation/perfusion
